# The Prevalence of Polycystic Ovary Syndrome, Its Phenotypes and Cardio-Metabolic Features in a Community Sample of Iranian Population: Tehran Lipid and Glucose Study

**DOI:** 10.3389/fendo.2022.825528

**Published:** 2022-03-01

**Authors:** Mahbanoo Farhadi-Azar, Samira Behboudi-Gandevani, Maryam Rahmati, Fatemeh Mahboobifard, Ensi Khalili Pouya, Fahimeh Ramezani Tehrani, Fereidoun Azizi

**Affiliations:** ^1^ Reproductive Endocrinology Research Center, Research Institute for Endocrine Sciences, Shahid Beheshti University of Medical Sciences, Tehran, Iran; ^2^ Faculty of Nursing and Health Sciences, Nord University, Bodø, Norway; ^3^ Department of Pharmacology, School of Medicine, Shahid Beheshti University of Medical Sciences, Tehran, Iran; ^4^ Faculty of Medicine, Shahid Beheshti University of Medical Science, Tehran, Iran; ^5^ Endocrine Research Center, Research Institute for Endocrine Sciences, Shahid Beheshti University of Medical Sciences, Tehran, Iran

**Keywords:** cardio-metabolic disturbances, Tehran Lipid and Glucose Study, polycystic ovary syndrome, prevalence, metabolic syndrome

## Abstract

**Objectives:**

The aim of the present study was to evaluate the prevalence of polycystic ovary syndrome (PCOS), its phenotypical and cardio-metabolic features in a community sample of the Iranian population in comparison to healthy eumenorrheic, non-hirsute women without polycystic ovaries. The second aim was to assess the cardio-metabolic characteristics of women who suffered from one criteria of PCOS compared to those healthy eumenorrheic, non-hirsute women.

**Methods:**

In this cross-sectional population-based study, a total of 1,960 eligible women, aged (18–45 years) were recruited from the Tehran-Lipid and Glucose-Study participants and were classified as the three groups of (i) women with PCOS by the Rotterdam criteria, (ii) non-PCOS women with one criteria of PCOS and (iii) healthy eumenorrheic, non-hirsute women without polycystic ovaries morphology (PCOM) as the control group. Further PCOS women were extended to four phenotypes of hyperandrogenism, oligo-anovulation, polycystic ovaries (phenotype A), hyperandrogenism, oligo/anovulation (phenotype B), hyperandrogenism, polycystic ovaries (phenotype C) and oligo-anovulation, polycystic ovaries (phenotype D). Cardio-metabolic profiles and the prevalence of comorbidities of metabolic syndrome (MetS) and lipid abnormalities were compared among these groups linear, and the median regression models adjusted for age and body mass index.

**Results:**

The prevalence of PCOS according to the diagnostic criteria of the NIH, Rotterdam and AE-PCOS Society were 13.6, 19.4, and 17.8, respectively. Among those who met the Rotterdam criteria, 23.9, 46.3, 21.6, and 8.2% had phenotypes A, B, C, and D, respectively. Among the remaining 1,580 women who did not fulfil the PCOS criteria, 108 (6.8%) suffered from only oligo/anovulation, 332 (21%) only hyperandrogenism/hyperandrogenemia, 159 (16.2%) only PCOM in ultrasound and 981 (62%) were healthy eumenorrheic, non-hirsute women without PCOM. The study revealed that some adiposity indices and lipid abnormalities in PCOS phenotypes with hyperandrogenism (A, B, and C) were worse than in healthy women. By contrast, women with phenotype D did not differ from the healthy ones in terms of adiposity and lipid abnormalities. However, the respective values for other cardio-metabolic profiles and MetS rates in different phenotypes of PCOS were similar to the healthy women. Only the prevalence of MetS in phenotype A was significantly higher than in the healthy women. There were no statistically significant differences between participants with one criteria of PCOS and healthy counterparts in terms of most adiposity indexes, cardio-metabolic factors, and comorbidity of MetS and its components. However, women with hyperandrogenism had a significantly higher level of the waist to height ratio (WHtR) and hypertriglyceridemia than their healthy counterparts.

**Conclusion:**

PCOS, mainly classical phenotypes A and B, are common among Iranian women of reproductive age. Women with PCOS who had androgen excess exhibited the worst lipid profile, and those who had full three criteria of the syndrome exhibited the higher rate of MetS. However, women with only ovulatory dysfunction and only PCOM had similar cardio-metabolic characteristics, compared to healthy subjects. These data suggest that routine screening for metabolic disturbances may be needed in the prevention of cardio-metabolic disorders in patients with more serious phenotypes of PCOS.

## Introduction

Polycystic ovary syndrome (PCOS) is the common endocrine disturbance worldwide, affecting 6–12% of women of reproductive age (1). The syndrome is a complex and heterogeneous disorder that may have various clinical manifestations, mainly including hyperandrogenism and/or hyperandrogenemia (HA), oligo/anovulation (OA) and polycystic ovaries morphology (PCOM) (2, 3). Although the exact underlying etiology of PCOS remains unclear, emerging evidence suggests that insulin resistance (IR) appears to be implicated in its pathogenesis (4–6).

However, the results of studies focusing on the rate of the syndrome in the general population are insufficient and vary in different populations. Using different diagnostic criteria of PCOS, namely, the National Institutes of Health (NIH) (7), the 2003 Rotterdam (8, 9), and the Androgen Excess Society (AES) (10) definitions could potentially affect the estimation of PCOS prevalence (11). According to the Rotterdam criteria, women with PCOS can be divided into 4 phenotypes (12). Additionally, the lack of standard approaches in diagnostic elements within each set of criteria, such as diagnosing oligo/anovulation and androgen excess and a technical issue in PCOM assessment, potentially impacts prevalence estimates of PCOS (13). However, the handling of systemic hormonal contraception and its effect on PCOS manifestation is another issue that should take account.

At present, some abnormalities in metabolic pathways such as insulin signaling and steroid hormone regulation pathways have been proposed in the underlying pathophysiology of PCOS (14). However, the results of studies focusing on cardio-metabolic disturbances among women with PCOS are controversial (15–18). These challenges stem from the fact that some of the current evidence is usually based on clinical studies, mainly with a small sample population that may not recruit a milder form of PCOS (19). Additionally, there are significant ethnic, racial and geographical variations in the clinical manifestation of PCOS and its metabolic characteristics (20).

Moreover, some studies reported an increase in metabolic disturbances among women who meet only one criteria of PCOS (21–24), whereas some other studies did not confirm such findings (25, 26).

Due to a lack of adequate population-based evidence, this study aimed to investigate the prevalence of PCOS, and its phenotypical. Cardio-metabolic features compared to healthy eumenorrheic, non-hirsute women without polycystic ovaries in a community-based study Tehran Lipid and Glucose Study (TLGS). The second aim was to assess the cardio-metabolic characteristics of women who suffered from one criteria of PCOS compared to those healthy eumenorrheic, non-hirsute women without polycystic ovaries in those populations.

## Materials and Methods

The participants of the present study were recruited from the TLGS. This study is a prospective, long-term, community-based cohort study started in 1998, mainly aimed to evaluate the factors associated with non-communicable disorders in a representative sample of Tehran, Iran. Details of the TLGS have been presented elsewhere (27). The study was approved by the ethical review board of the Research Institute for Endocrine Sciences, Shahid Beheshti University of Medical Sciences, Tehran, Iran (Code: IR.SBMU.ENDOCRINE.REC.1398.070) and informed consent was obtained from all participants.

### Study Population

For the current study, data collected in the third follow-up visit of the TLGS (2005–2008) was used. It included comprehensive data on participants’ obstetrics and reproductive history (28). To avoid underestimating the PCOS, we recruited all women aged 18–45 years, regardless of using medications, namely, those taking glucocorticoid, an insulin sensitizer, anti-androgen therapy, oral contraceptive pills or continuous progestin prevalence. In addition, we assessed all medical records, namely, medical documents, drugs, laboratory and ultrasound assessments, to confirm self-report information.

Pregnant and menopausal women, those with a history of endocrine disorders, namely, thyroid disease, congenital adrenal hyperplasia, hyperprolactinemia, Cushing’s syndrome, and androgen-secreting neoplasm were excluded from the study. Finally, the remaining participants were classified as PCOS, women with only one criteria of PCOS, namely only OA, Only HA, only PCOM and healthy eumenorrheic, non-hirsute women without PCOM. Further PCOS women were extended to four phenotypes of A (namely, HA + OA + PCOM), B (namely, OA + HA), C (namely, HA + PCOM) and D (OA+ PCOM).

All general anthropometric and physical examinations were performed by a general practitioner. For biochemical measurement, all blood samples were taken between 7:00 and 9:00 AM after 12 h of overnight fasting during the early follicular phase of the spontaneous or progesterone-induced menstrual cycle. All anthropometric and biochemical measurements were published elsewhere (27–30) and presented in **Supplementary File 1**. It should be noted that in those with confirmed PCOS that used hormones, they were asked about their menstrual pattern, acne and hirsutism status before taking those medications. In addition, the hormonal assessment did not perform among them.

## Definition of Terms

We defined PCOS in our study using the (i) Rotterdam criteria (8, 9), as the presence of two or more of the following criteria, namely, oligo/anovulation, clinical or biochemical hyperandrogenism and polycystic ovaries morphology, (ii) the National Institutes of Health Criteria (NIH) (7) which defines as the presence of two of the following criteria namely, oligo/anovulation, clinical or biochemical hyperandrogenism, and (iii) the Androgen Excess—Polycystic Ovary Syndrome (AE-PCOS) Society (10), which includes women with clinical or biochemical hyperandrogenism and oligo/anovulation or polycystic ovaries on ultrasound. Oligo/anovulation was defined as either regular or irregular menstrual cycles ≥34 days or those who had a history of eight or fewer menstrual cycles in a year. The clinical manifestations of hyperandrogenism included hirsutism diagnosed based on a standardized scoring system of modified Ferriman–Gallwey scale (≥8) (31), acne, or androgenic alopecia. Acne was assessed quantitatively based on its type, number and distribution (32). Biochemical hyperandrogenism was assessed as an increased level of one or more serum androgens—namely, dehydroepiandrosterone sulfate, testosterone, or androstenedione—above the 95th percentile, determined in the selected healthy non-hirsute eumenorrheic women in the study population (33). In this respect, those upper limits of the normal upper limits were 0.89 ng/ml for TT, 2.9 ng/ml for A4; 179 mg/dl for DHEAS and 5.39 for FAI. PCOM was diagnosed by the presence of 12 or more follicles in each ovary, measuring 2–9 mm in diameter and/or increased ovarian volume more than 10 cm^3^) (33).

Adiposity indexes were calculated using the following formula: Body mass index (BMI) was calculated using the formula as the weight in kg divided by the square of height in meters (kg/m^2^). Waist circumference (WC) was measured using an unstretched tape midway between the inferior costal margin and the superior border of the iliac crest. The hip circumference (HC) was measured using an unstretched tape at the anterior superior iliac spine level without any pressure to the body surface. The waist to hip ratio (WHR) was calculated as WC divided by HC in cm. The waist to height ratio (WHtR) was calculated as WC in centimeters (cm) divided by height in cm. lipid accumulation product (LAP) = [WC (cm) − 58] × [TG concentration (mmol/L)] (34); female visceral adiposity index (VAI) = [WC(cm)/36.58 + (1.89 × BMI)] × (TG/0.81) × (1.52/HDL-C)]; a body shape index (ABSI) = [WC (cm)/BMI^2/3^ × height (m)^1/2^] (35).

We defined MetS according to the Joint Interim Statement criteria (36) as the presence of any three or more of the following five risk factors: [1] fasting triglycerides (TG) level of ≥150 mg/dl or specific treatment; [2] fasting high-density lipoprotein (HDL) ≤50 mg/dl or specific treatment; [3] raised systolic blood pressure (SBP) ≥130 mmHg, or raised diastolic blood pressure (DBP) ≥85 mm Hg, or receiving treatment; [4] fasting plasma glucose of ≥100 mg/dl or treatment, and [5] high waist circumference using waist-circumference cutoff points of >90 cm according to the population and the country-specific cutoff point for Iranians (37).

### Statistical Analysis

Normality distribution of continuous variables was assessed using the one-sample Kolmogorov–Smirnov test. Categorical variables were presented as numbers and percentages. The comparison of variables between groups was made by applying the two-independent sample t-test (or ANOVA) and *X* ^2^ test for continuous and categorical data, respectively. The Mann–Whitney U test (or Kruskal–Wallis test) was applied to compare variables with skewed distribution. Linear or median regression models were applied for continuous and logistic regression for categorical data for calculating age-BMI adjusted p-values as appropriate for between-group comparisons. Those using medications that affected the hormonal levels were excluded from the relevant analysis. Statistical analysis was performed using software package STATA (version 13; STATA Inc., College Station, TX, USA); significance level was set at P <0.05.

## Results

**Figure 1:**
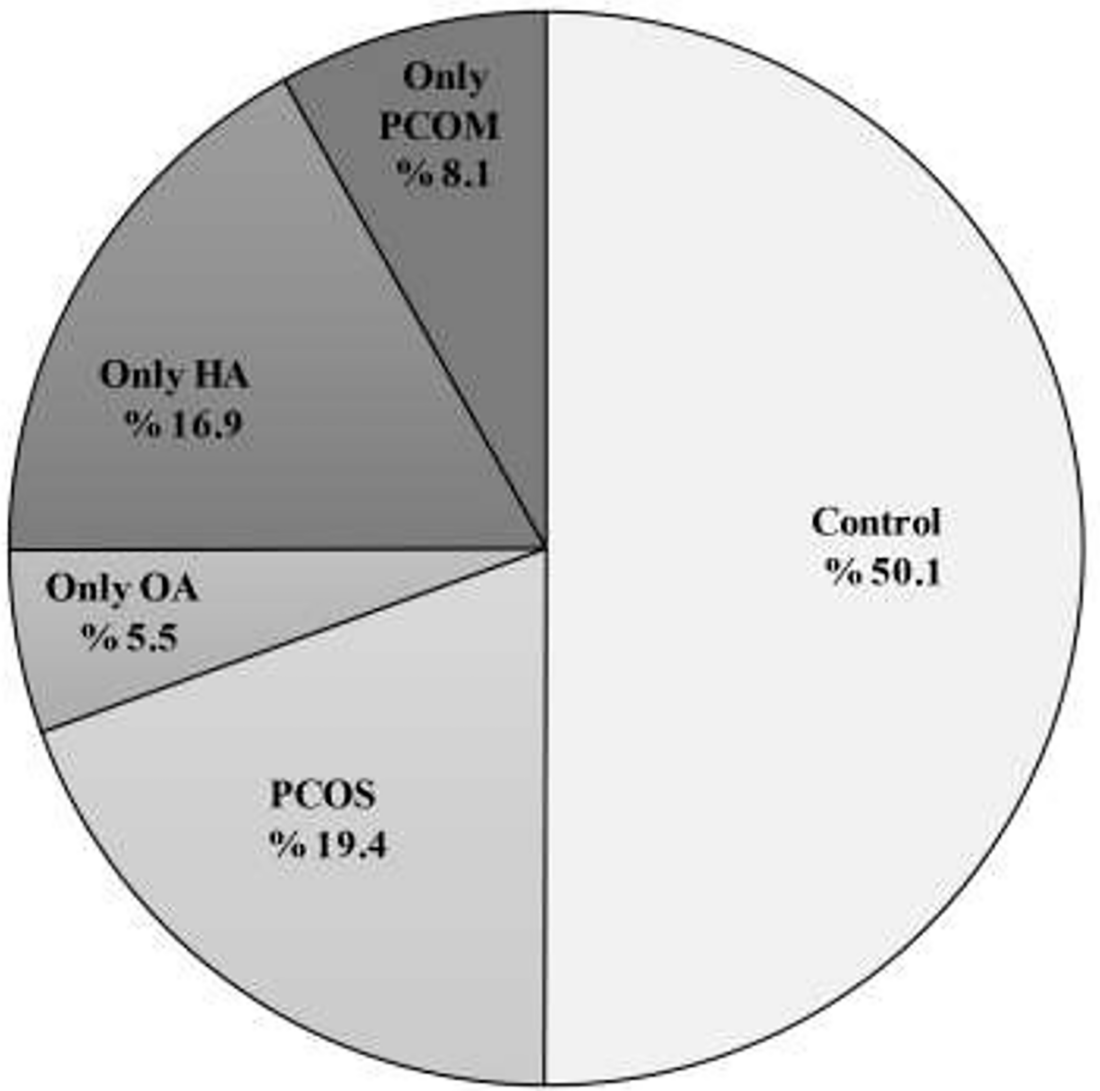
Distribution (%) of PCOS women patients with phenotypes A, B, C, and D and also women with only OA, HA, and PCOM with the control group. PCOS, Polycystic ovary syndrome; OA, oligo anovulation; HA, hyperandrogenism. PCOM, polycystic ovaries morphology.

A total of 1,960 participants were eligible and included in the study. **Figure 1** shows the distribution of the study population based on different groups.

The prevalence of PCOS according to the diagnostic criteria of NIH, Rotterdam and AE-PCOS Society were 13.6% (267/1,960), 19.4% (380/1,960), and 17.8 (349/1,960), respectively. Among those who met the Rotterdam criteria for PCOS, the proportion of PCOS phenotype was 23.9% (91/380) for phenotype A (namely, OA, HA, PCOM), 46.3% (176/380) for phenotype B (namely, OA, HA), 21.6% (82/380) for phenotype C (namely, HA, PCOM), and 8.2 (31/380) for phenotype D (namely, OA, PCOM), respectively. Among the remaining 1,580 women who did not fulfill the PCOS criteria, 108 (6.8%) suffered from only oligo/anovulation, 332 (21%) only hyperandrogenism/hyperandrogenemia, 159 (16.2%) only PCOM in ultrasound and 981 (62%) were healthy eumenorrheic, non-hirsute women without PCOM (**Figure 1**).

**Table 1** shows the features in women with PCOS and healthy counterparts. Compared to healthy women, women with PCOS were more likely to be younger (31.1 (7.9) versus 32.4 (7.6) years (P = 0.002)) and showed significantly higher serum levels of total testosterone (TT) (0.6 (0.3–0.8) vs. 0.3 (0.1–0.6) ng/dl; P <0.001), androstenedione (A4) (1.8 (1.0–2.4) vs. 1.1 (0.9–1.7) ng/ml; P <0.001), DHEAS (160.5 (93.7–212.5) vs. 124.8 (67.9–179.3) μg/dl; P <0.001), free androgen index (FAI) (1.2 (0.6–2.0) vs. 0.5 (0.2–1.0);P >0.001), versus healthy ones (**Table 1**). After adjustment for potential confounders of age and BMI, there were no significant differences in mean values of BMI, WC, HC, WHR, WHtR, and ABSI between PCOS and healthy subjects. The cardio-metabolic profile of women with PCOS, namely, FBG, 2-hour plasma glucose (Bs-2hPG), TC, TG, LDL-C, SBP, and DBP, were comparable with healthy women. The prevalence of comorbidity of MetS and dyslipidemia among women with PCOS were similar to healthy women. The distribution of components of the MetS was also similar in PCOS and healthy women, only the rate of hypertriglyceridemia and hypo-HDL-cholesterolemia in women with PCOS were significantly higher than healthy subjects (25.8% vs. 19.1%, P <0.001) and (36.9% vs. 28.4%, P = 0.003), respectively (**Table 1**).

**Table 1:** Comparison the population characteristics of women with PCOS and healthy women.

	PCOS (n = 380)	Healthy women (n = 981)	Unadjusted P-value^¥^	Age and BMI Adjusted P-value^€^
Age, years, mean (SD)	31.1 (7.9)	32.4 (7.6)	0.01	0.002
Adiposity index, mean (SD) or median (25–75%)				
BMI, (kg/m^2)^	26.23 (5.0)	26.20 (4.6)	0.772	0.119
HC, cm	101.1 (8.9)	101.2 (8.6)	0.902	0.307
WC, cm	81.7 (12.1)	81.8 (11.7)	0.921	0.995
WHR	0.81 (0.1)	0.81 (0.1)	0.949	0.648
WHtR	0.51 (0.1)	0.52 (0.1)	0.632	0.383
ABSI	7.3 (7.0–7.7)	7.4 (7.0–7.7)	0.214	0.601
VAI	99.7 (57.9–183.2)	87.0 (54.9–147.1)	**0.021**	**0.041**
LAP	26 (12.0–53.2)	24.2 (13.0–44.2)	0.305	**0.012**
# Hormones, mean (SD) or median (25–75%)				
LH, (mIU/ml)	5.9 (3.8–9.2)	4.7 (3.4–6.4)	<0.001	**0.002**
FSH, (mIU/ml)	7.0 (5.2–9.1)	7.6 (5.6–10)	0.002	**0.022**
17 OH-P, (nmol/L)	1.6 (0.9–2.4)	1.3 (1.0–2.1)	0.265	0.673
TT, ng/dl	0.6 (0.3–0.8)	0.3 (0.1–0.6)	<0.001	**<0.001**
FAI	1.2 (0.6–2.0)	0.5 (0.2–1.0)	<0.001	**<0.001**
A4, ng/ml	1.8 (1.0–2.4)	1.1 (0.9–1.7)	<0.001	**<0.001**
DHEAS, μg/DI	160.5 (93.7–212.5)	124.8 (67.9–179.3)	<0.001	**<0.001**
SHBG, nmol/L	44.9 (33.4–61.0)	59.7 (44.4–84.4)	<0.001	**<0.001**
# Cardio-metabolic factors, mean (SD) or median (25–75%)				
FBG (mmol/L)	4.7 (4.4–5.0)	4.7 (4.4–5.0)	0.734	0.504
Bs-2hPG (mmol/L)	5.2 (4.4–6.2)	5.1 (4.4–5.9)	0.416	0.575
TC (mmol/L)	4.4 (3.9–5.0)	4.5 (3.9–5.1)	0.668	0.554
TG (mmol/L)	1.1 (0.8–1.8)	1.1 (0.8–1.5)	**0.025**	0.078
HDL-C (mmol/L)	1.1 (0.9–1.3)	1.2 (1.0–1.3)	**0.005**	**0.035**
LDL-C (mmol/L)	2.6 (2.1–3.2)	2.7 (2.2–3.2)	0.304	0.109
SBP (mmHg)	103.6 (12.7)	103.6 (12.1)	0.926	0.903
DBP (mmHg)	68.9 (9.4)	68.8 (9.1)	0.908	0.629
# Comorbidities, n (%)				
Metabolic Syndrome	58 (15.5)	122 (12.7)	0.215	0.066
WC >90, (cm)	87 (24)	194 (20.8)	0.238	0.216
SBP ≥130 (mmHg)	5 (1.3)	28 (2.9)	0.192	0.354
DBP ≥85 (mmHg)	18 (4.8)	38 (3.9)	0.564	0.447
FBS ≥6.1 (mmol/L)	6 (1.6)	31 (3.2)	0.112	0.208
HDL-C <1.03 (mmol/L)	140 (36.9)	274 (28.4)	**0.002**	**0.003**
Lipids disturbances, n (%)				
TG ≥1.7 (mmol/L)	98 (25.8)	184 (19.1)	**0.006**	**0.001**
TC ≥5.02 (mmol/L)	99 (26.0)	252 (26.1)	0.915	0.736
LDL-C ≥3.04 (mmol/L)	112 (31.3)	312 (34.6)	0.382	0.318

Values are presented as mean (SD), median (25–75%) or number (percentage) as appropriate.

#Regardless of those taking oral contraceptive pills, continuous progestin, glucocorticoid, or insulin sensitizer or anti androgen therapy (n = 85 including 63 in PCOS group and 22 in Healthy women).

^¥^P-value is calculated by two-independent sample t-test or Mann–Whitney U test for continuous, and x^2^ test for categorical data as appropriate for between group comparisons.

^€^Adjusted p-value is calculated by linear regression or median regression for continuous and logistic regression for categorical data as appropriate for between group comparisons. Bold values indicate statistical significance.

PCOS, Polycystic ovary syndrome; OA, oligo anovulation; HA, hyperandrogenism; PCOM, polycystic ovaries morphology; BMI, Body mass index; HC, hip circumference; WC, Waist circumference; WHR, waist to hip ratio; WHtR, waist to height ratio; ABSI, a body shape index; VAI, visceral adiposity index; LAP, lipid accumulation product. LH, luteinizing hormone; FSH, follicle stimulating hormone; 17OH-P, 17-hydroxyprogesterone; TT, Total testosterone; FAI, free androgen index; A4, androstenedione; DHEAS, dehydroepiandrosterone sulfate; SHBG, sex hormone binding globulin; FBG, fasting blood glucose; Bs-2hPG, 2-hour plasma glucose; TC, total cholesterol; TG, triglycerides; HDL, high-density lipoprotein; LDL, low-density lipoprotein; SBP, systolic blood pressure; DBP, diastolic blood pressure.

**Table 2** shows the features of the participants, classified into 4 phenotypical groups of PCOS and healthy women.

**Table 2:** Features of the participants, classified to 4 phenotypical groups of PCOS and healthy women.

	PCOS Phenotype A(OA + HA + PCOM)(n = 91)	PCOS Phenotype B(OA + HA)(n = 176)	PCOS Phenotype C(HA + PCOM)(n = 82)	PCOS Phenotype D(OA + PCOM)(n = 31)	Healthy women(n = 981)*
Age, year, mean (SD)	31.5 (7.4)	31.1 (8.0)	30.8 (8.1)	30.8 (8.3)	32.4 (7.6)^b^
Adiposity index, mean (SD) or median (25–75%)					
BMI, (kg/m^2^)	26.4 (4.4)	26.6 (5.5)	25.8 (4.8)	25.3 (4.0)	26.2 (4.6)^b^
HC, cm	101.0 (8.6)	102.0 (9.7)	100.1 (8.4)	99.2 (7.5)	101.2 (8.6)
WC, cm	82.3 (11.5)	82.1 (13.0)	81.0 (11.5)	79.7 (10.4)	81.8 (11.7)
WHR	0.81 (0.07)	0.80 (0.09)	0.81 (0.07)	0.80 (0.07)	0.81 (0.07)
WHtR	0.52 (0.07)	0.52 (0.09)	0.51 (0.08)	0.50 (0.07)	0.52 (0.07)
ABSI	7.3 (7.0–7.7)	7.3 (6.9–7.7)	7.3 (7.1–7.7)	7.3 (7.0–7.7)	7.37 (7.03–7.7)
VAI	118.8 (60.3–213.1)	98.4 (57.8–175.5)	89.0 (54.4–156.6)	83.2 (60.3–156.9)	87.0 (54.9–147.1)^a^ ^,^ ^b^
LAP	30.6 (13.8–60.9)	26.7 (11.0–48.6)	19.9 (12.5–43.3)	24.2 (11.3–44.9)	24.2 (13.0–44.2)
#Hormones, mean (SD) or median (25–75%)					
LH, mIU/ml	4.7 (3.1–8.8)	6.3 (3.8–9.2)	6.8 (4.3–10)	4.9 (3.7–6.9)	4.7 (3.4–6.4)^b^ ^,^ ^c^
FSH, mIU/ml	6.8 (4.8–9.1)	7.3 (5.4–9.1)	7.5 (5.3–9.2)	6.9 (5.3–9.0)	7.6 (5.6–10)^a^ ^,^ ^d^
17 OH-P, nmol/L	1.3 (0.8–2.1)	1.7 (1.1–2.7)	1.9 (1.4–2.4)	1.5 (1.1–2.2)	1.3 (1.0–2.1)
TT, ng/dl	0.5 (0.3–0.8)	0.5 (0.3–0.8)	0.5 (0.3–0.8)	0.4 (0.3–0.7)	0.3 (0.1–0.5)^a^ ^,^ ^b^ ^,^ ^c^ ^,^ ^d^
FAI	1.2 (0.6–1.9)	1.3 (0.6–2.1)	1.0 (0.5–2.0)	0.8 (0.5–1.4)	0.5 (0.2–0.9)^a^ ^,^ ^b^ ^,^ ^c^ ^,^ ^d^
A4, ng/ml	1.6 (0.9–2.3)	1.8 (1.0–2.4)	1.9 (1.3–2.5)	1.8 (1.1–2.4)	1.1 (0.9–1.7)^a^ ^,^ ^b^ ^,^ ^c^
DHEAS, μg/DI	148 (85.1–225.5)	168.2 (101.2–207.8)	150 (90.7–213.3)	179.7 (118.3–205.5)	124.9 (67.9–179.3)^b^ ^,^ ^c^ ^,^ ^d^
SHBG, nmol/L	42.9 (31.5–62.5)	43.6 (31.7–55.6)	45.5 (33.6–55.8)	62.1 (46.8–72.5)	59.7 (44.4–84.4)^a^ ^,^ ^b^ ^,^ ^c^
#Cardio-metabolic factors, mean (SD) or median (25–75%)					
FBG (mmol/L)	4.7 (4.4–5.0)	4.7 (4.5–5.0)	4.7 (4.4–5.0)	4.7 (4.4–4.9)	4.7 (4.4–5.0)
Bs-2hPG ;(mmol/L)	5.6 (4.4–6.4)	5.3 (4.4–6.1)	5.0 (4.1–5.9)	5.0 (4.4–5.7)	5.1 (4.4–5.9)
TC (mmol/L)	4.5 (3.9–5.2)	4.4 (3.9–5.2)	4.3 (3.8–4.9)	4.2 (3.8–5.0)	4.5 (3.9–5.1)
TG (mmol/L)	1.4 (0.8–2.2)	1.1 (0.8–1.6)	1.0 (0.8–1.4)	1.2 (0.7–1.8)	1.0 (0.8–1.5)^b^
HDL-C (mmol/L)	1.1 (0.9–1.4)	1.1 (0.9–1.3)	1.1 (0.9–1.2)	1.1 (0.9–1.3)	1.2 (1.0–1.3)^c^
LDL-C (mmol/L)	2.6 (2.1–3.1)	2.7 (2.1–3.3)	2.6 (2.2–3.1)	2.5 (2.2–3.1)	2.7 (2.2–3.2)
SBP (mmHg)	103.4 (15.5)	104 (12)	102.8 (11.6)	103.8 (10.8)	103.5 (12.1)
DBP (mmHg)	68 (9.5)	69.7 (10.1)	68.6 (7.9)	68.1 (7.9)	68.8 (9.1)
#Comorbidities, n (%)					
Metabolic Syndrome	17 (19.3)	25 (14.4)	12 (15.0)	4 (12.9)	122 (12.7)^a^
WC >90	23 (26.1)	44 (26.2)	15 (19.5)	5 (16.7)	194 (20.8)
SBP ≥130	1 (1.1)	2 (1.1)	2 (2.5)	0	28 (2.9)
DBP ≥85	3 (3.3)	12 (6.9)	2 (2.5)	1 (3.2)	38 (3.9)
FBS ≥6.1	2 (2.2)	3 (1.7)	1 (1.2)	0	31 (3.2)
HDL-C <1.03	30 (33)	66 (37.7)	33 (40.2)	11 (35.5)	274 (28.4)^b^ ^,^ ^c^
Lipids disturbances					
TG ≥1.7	34 (37.4)	40 (22.7)	16 (19.5)	8 (25.8)	184 (19.1)^a^
TC ≥5.02	28 (30.8)	48 (27.3)	16 (19.5)	7 (22.6)	252 (26.1)
LDL-C ≥3.04	23 (27.1)	58 (34.3)	19 (25.3)	12 (41.4)	312 (34.6)

Those using various medications were excluded from relevant analysis.

#Regardless of those taking oral contraceptive pills, continuous progestin, glucocorticoid, or insulin sensitizer or anti androgen therapy (n = 85 including 63 in PCOS group and 22 in Healthy women).

*Adjusted p-value is calculated by linear regression or median regression for continuous and logistic regression for categorical data as appropriate for between group comparisons.

a P < 0.05 for differences between individuals with PCOS Phenotype A and healthy women.

b P < 0.05 for differences between individuals with PCOS Phenotype B and healthy women.

c P < 0.05 for differences between individuals with PCOS Phenotype C and healthy women.

d P < 0.05 for differences between individuals with PCOS Phenotype D and healthy women.

PCOS, Polycystic ovary syndrome; OA, oligo anovulation; HA, hyperandrogenism; PCOM, polycystic ovaries morphology; BMI, Body mass index; HC, hip circumference; WC, Waist circumference; WHR, waist to hip ratio; WHtR, waist to height ratio; ABSI, a body shape index; VAI, visceral adiposity index; LAP, lipid accumulation product. LH, luteinizing hormone; FSH, follicle stimulating hormone; 17OH-P, 17-hydroxyprogesterone; TT, Total testosterone; FAI, free androgen index; A4, androstenedione; DHEAS, dehydroepiandrosterone sulfate; SHBG, sex hormone binding globulin; FBG, fasting blood glucose; Bs-2hPG, 2-hour plasma glucose; TC, total cholesterol; TG, triglycerides; HDL, high-density lipoprotein; LDL, low-density lipoprotein; SBP, systolic blood pressure; DBP, diastolic blood pressure.

The study revealed that some adiposity indices and lipid abnormalities in PCOS phenotypes with hyperandrogenism (A, B, and C) were worse than in healthy women. As such, women with PCOS-phenotype A had a higher level of VAI (118.8 (60.3–213.1) vs. 87.0 (54.9–147.1)), women with phenotype B had significantly higher BMI (26.6 (5.5) vs. 26.2 (4.6) kg/m^2^), serum level of TG (1.1 (0.8–1.6) vs. 1.0 (0.8–1.5) mmol/L) and those with phenotype C had lower serum level of HDL-C (2.6 (2.2–3.1) vs. 2.7 (2.2–3.2) mmol/L) than healthy counterparts. By contrast, women classified as phenotype D did not differ from healthy ones in terms of adiposity and lipid abnormalities. However, the respective values for other cardio-metabolic profiles in different phenotypes of PCOS were similar to healthy women. The rates of MetS were 17% (17/91) for phenotype A, 14.4% (25/176) for phenotype B, 15% (12/82) phenotype C, and 12.9% (4/31) for phenotype D. Regression analysis showed that after adjustment for age and BMI, only the prevalence of MetS in phenotype A was significantly higher than in the healthy women (19.3% vs. 12.7%, P-value = 0.02). Further, we found that TT levels observed in phenotype D were within the normal range but still higher than women with a normal population (0.4 (0.3–0.7) vs. 0.3 (0.1–0.5), ng/dl, P-value = 0.001), respectively.

**Table 3** shows the features of the study characteristics classified those with one criteria of PCOS groups compared to healthy women. Age and BMI adjusted regression models revealed no statistically significant differences between participants with one criteria of PCOS and healthy counterparts in terms of most adiposity indexes, cardio-metabolic factors and comorbidity of MetS and its components. However, women with only hyperandrogenism had significantly higher level of WHtR (0.5 (0.08) vs. 0.5 (0.07), P-value = 0.02) and hypertriglyceridemia (29.5% vs. 21.4%, P-value = 0.02) than healthy counterparts (**Table 3**). As we expected, women with only hyperandrogenism had higher androgenic hormones concentration, namely, TT, FAI, A4, and DHEAS, compared to healthy ones. However, the level of those hormones in women with only OA and PCOM were comparable with healthy participants. TT level observed in women with PCOM were within the normal range but still higher than women with normal population (0.3 (0.3–0.4) vs. 0.3 (0.1–0.6), ng/dl, P-value = 0.01), respectively.

**Table 3:** Features of the study characteristics classified to PCOS or those with one criteria of PCOS groups and healthy women.

	Only OAn = 108	Only HAn = 332	Only PCOMn = 159	Healthy women *n = 981
Age, years, mean (SD)	35.7 (7.3)	31.8 (8.0)	28.8 (7.1)	32.4 (7.6) ^€,£^
Adiposity index, mean (SD) or median (25–75%)				
BMI, (kg/m^2^)	27.8 (4.7)	25.6 (4.7)	25.5 (5.0)	26.2 (4.6)
HC, cm	103.1 (9.3)	100.5 (8.8)	100.0 (9.0)	101.2 (8.6)
WC, cm	84.8 (11.7)	80.2 (11.7)	79.3 (12.2)	81.8 (11.7)
WHR	0.8(0.07)	0.7(0.07)	0.7 (0.07)	0.8 (0.07)
WHtR	0.5 (0.08)	0.5 (0.08)	0.5 (0.08)	0.5 (0.07) ^§^
ABSI	7.4 (7.1–7.6)	7.3 (6.9–7.7)	7.3 (6.9–7.6)	7.4 (7.0–7.7)
VAI	113.8 (75.6–180.1)	85.2 (54.2–148.6)	88.9 (50.5–141)	87 (54.9–147.1)
LAP	24.2 (18.8–54.3)	22.1 (11.1–43.2)	20.9 (10.1–37.1)	24.2 (13–44.2)
#Hormones, mean (SD) or median (25–75%)				
LH, mIU/ml	5.5 (3.6–7.5)	4.7 (3.2–6.8)	5.4 (3.6–7.4)	4.7 (3.4–6.4)
FSH, mIU/ml	7.7 (5.1–10.6)	8.4 (6.1–10.7)	7.4 (4.6–10.9)	7.6 (5.6–10) ^§^
17 OH-P, nmol/L	1.6 (1.2–2.7)	1.8 (1.2–2.8)	1.2 (0.9–1.8)	1.3 (1–2.1) ^§^
TT, ng/dl	0.4 (0.2–0.6)	0.4 (0.2–0.6)	0.3 (0.3–0.4)	0.3 (0.1–0.6) ^§,£^
FAI	0.7 (0.3–1.0)	0.8 (0.3–1.5)	0.9 (0.4–2.6)	0.5 (0.2–1.0) ^§^
A4, ng/ml	1.6 (1–2)	1.6 (1–2.2)	0.8 (0.2–2.2)	1.1 (0.9–1.7) ^§^
DHEAS, μg/DI	142.8 (76.7–192.2)	168 (91–229)	120 (89–190)	124.9 (67.9–179.3) ^§^
SHBG, nmol/L	59 (48.3-87.3)	48.2 (31.5–64)	33 (15–68)	59.7 (44.4–84.4) ^§^
#Cardio-metabolic factors, mean (SD) or median (25–75%)				
FBG (mmol/L)	4.8 (4.5–5.0)	4.7 (4.4–4.9)	4.7 (4.4–4.8)	4.7 (4.5–5.0)
BS (mmol/L)	5.2 (4.5–6.0)	5 (4.3–5.9)	5.1 (4.4–5.8)	5.1 (4.4–6.0)
TC (mmol/L)	4.6 (4.1–5.2)	4.4 (3.9–5.0)	4.5 (3.8–5.0)	4.5 (3.9–5.1)
TG (mmol/L)	1.3 (1.0–1.8)	1.1 (0.8–1.6)	1.1 (0.8–1.6)	1.1 (0.8–1.5)
HDL-C (mmol/L)	1.2 (1.0–1.3)	1.2 (0.9–1.3)	1.2 (1.0–1.3)	1.2 (1.0–1.3)
LDL-C (mmol/L)	2.9 (2.4–3.3)	2.7 (2.2–3.2)	2.7 (2.2–3.1)	2.7 (2.2–3.2)
SBP (mmHg)	106.2 (12.9)	102.9 (11.4)	103.1 (11.9)	103.6 (12.1)
DBP (mmHg)	71.6 (8.0)	68.2 (9.2)	67.5 (9.5)	68.8 (9.1)
#Comorbidities, n (%)				
Metabolic Syndrome	58 (15.5)	20 (19.4)	44 (13.6)	20 (12.9)
WC >90	33 (31.4)	61 (19.1)	29 (19.1)	194 (20.8)
SBP ≥130	7 (6.7)	5 (1.5)	5 (3.2)	28 (2.9)
DBP ≥85	7 (6.7)	11 (3.4)	6 (3.9)	38 (3.9)
FBS ≥6.1	5 (4.8)	9 (2.8)	2 (1.3)	31 (3.2)
HDL-C <1.03	37 (35.2)	105 (32.2)	39 (24.5)	274 (28.4)
Lipids disturbances				
TG ≥1.7	31 (29.5)	72 (22.1)	34 (21.4)	184 (19.1) ^§^
TC ≥5.02	30 (28.6)	72 (22.1)	41 (25.8)	252 (26.1)
LDL-C ≥3.04	43 (43.9)	98 (31.6)	45 (30)	312 (34.6)

Those using various medications were excluded from relevant analysis.

#Regardless of those taking oral contraceptive pills, continuous progestin, glucocorticoid, or insulin sensitizer or anti androgen therapy (n = 85 including 63 in PCOS group and 22 in Healthy women).

*Adjusted p-value is calculated by linear regression or median regression for continuous and logistic regression for categorical data as appropriate for between group comparisons.

^€^P < 0.05 for differences between individuals with only OA and healthy women.

^§^P < 0.05 for differences between individuals with only HA and healthy women.

^£^P < 0.05 for differences between individuals with only PCOM and healthy women.

PCOS, Polycystic ovary syndrome; OA, oligo anovulation; HA, hyperandrogenism; PCOM, polycystic ovaries morphology; BMI, Body mass index; HC, hip circumference; WC, Waist circumference; WHR, waist to hip ratio; WHtR, waist to height ratio; ABSI, a body shape index; VAI, visceral adiposity index; LAP, lipid accumulation product. LH, luteinizing hormone; FSH, follicle stimulating hormone; 17OH-P, 17-hydroxyprogesterone; TT, Total testosterone; FAI, free androgen index; A4, androstenedione; DHEAS, dehydroepiandrosterone sulfate; SHBG, sex hormone binding globulin; FBG, fasting blood glucose; Bs-2hPG, 2-hour plasma glucose; TC, total cholesterol; TG, triglycerides; HDL, high-density lipoprotein; LDL, low-density lipoprotein; SBP, systolic blood pressure; DBP, diastolic blood pressure.

## Discussion

This large population-based study in the Eastern Mediterranean Region revealed that PCOS prevalence was 19.4% by Rotterdam and phenotype B, namely, oligo/anovulatory dysfunction and hyperandrogenism, was the most prevalent phenotype of PCOS among the Iranian population. Regardless of age and BMI, lipid abnormalities such as low HDL-C and high TG levels were observed among women with PCOS, mainly in phenotypes with hyperandrogenism (A, B, and C), and other cardio-metabolic risk factors were comparable between PCOS and healthy counterparts. In addition, one-third of our population (30.5%) suffered from only one of the criteria for PCOS. Notably, whereas some abnormal anthropometric and hypertriglyceridemia were observed among women with only hyperandrogenism, women with only ovulatory dysfunction and only PCOM had similar cardio-metabolic characteristics compared to healthy subjects.

Despite the heavy burden of PCOS in different aspects of women’s health, few population-based representative surveys have been conducted, reporting the variation in prevalence rate from 6 to 22.5% by the Rotterdam criteria (11, 38–42). Further, there are still limited data concerning the particular phenotypes of PCOS, especially in different ethnic populations. However, in accordance with earlier studies conducted in Iran (11, 42, 43), results of our population-based study suggested PCOS, mainly classical phenotypes A and B, are common among Iranian reproductive-aged women. Our study is comparable with those few population-based studies that estimated PCOS prevalence by Rotterdam criteria, which were reported to be 8.5% in Brazil (41), 8.2% in China (40) and 22.5% in India (39). It is argued that ethnicity, racial, and geographical differences, the various definitions of each diagnostic criteria used such as hyperandrogenism, different PCOM manifestations and intervals of menstrual cycles as a criterion for oligo/anovulation, may affect the estimation of PCOS prevalence. Apart from those factors, these discrepancies also could be attributed to including adolescents or hormonal contraceptive users in the study population (1, 44).

However, much effort has been made to investigate the cardio-metabolic characteristics of women with PCOS, which have conflicting results. However, most of them derived from a clinical-based setting, with small sample sizes and lack of proper comparison control group without appropriate adjustment for potential confounders. In this community-based study, with appropriate sample size, we found that lipid abnormalities among women with PCOS who suffer from hyperandrogenism were more prevalent when compared to a healthy population. In agreement with these findings, a similar pattern of dyslipidemia has been described in women with the hyperandrogenic phenotype of PCOS (3, 14, 45–51). Further, Wild et al. in a meta-analysis, reported that women with PCOS have higher LDL-C and non-HDL-C, regardless of BMI (52). In this respect, testosterone and other androgenic hormones may lead to disturbed metabolic signaling pathways (53, 54).

Moreover, we found that hypertriglyceridemia is more prevalent in women with androgen excess. In agreement with this finding, Vonica et al. reported the significantly elevated levels of TG in women with PCOS compared with healthy, age-matched controls (55). Although hypertriglyceridemia may occur secondary to conditions such as glucose intolerance and central obesity in PCOS, however, it is shown that the androgen excess may increase the formation of free fatty acids and inflammatory cytokines, which *per se* is associated with hypertriglyceridemia. There is strong evidence that shows an association of higher triglycerides levels with IR, altered glucose tolerance and increased cardiovascular risk in the future (56). Moreover, hypertriglyceridemia has been demonstrated to be an important risk factor for cardiovascular disease. It could lead to an excess of triglyceride-rich lipoproteins (TRLs) and changes in the composition of key lipoprotein particles, including low- and high-density lipoproteins (LDL and HDL). This produces “atherogenic dyslipidemia,” with high TRLs, increased numbers of small, dense LDL, and low serum levels of HDL. It is also associated with increased pro-atherogenic particles and pro-inflammatory cytokines, which trigger the formation of a pro-oxidative, pro-atherogenic milieu (57, 58).

It is noteworthy that the prevalence of MetS among PCOS women was generally lower than in some other studies.

In two studies in Turkey, the prevalence of MetS in women between the ages of 40 and 50 were 36.7 and 22% (59, 60). In a meta-analysis, Hallajzadeh et al. (2018) reported that the pooled prevalence of MetS among PCOS women was 26.30% (95% CI: 23.68–28.93) but varied from 7.10% (95% CI: 1.64–12.56) to 37.50% (95% CI: 28.84–46.16), depending upon the diagnostic criteria used (61). Whereas these slight differences are probably due to genetic and environmental factors, however, the population-based study might include younger, lower BMI women with less severe phenotypes, which, taken together, may lead to a lower prevalence of MetS in the PCOS population. Besides, our findings also indicate that the prevalence of MetS in the PCOS and PCOS phenotypes B, C, and D are similar with a healthy population. However, its rate in phenotype A with concurrency of the three PCOS symptoms was higher in healthy women. Similar to our findings, other researchers have also reported the higher risk of MetS in frank PCOS phenotype A (62–64). However, the population-based setting of our study led us to recruit for the milder phenotype of PCOS, which may have a weaker association with IR and cardiovascular disease (15, 65).

In addition, endocrine profiles of women with only one PCOS criteria have been investigated in some studies (26, 66). In agreement with our findings, Rostami Dovom et al. (2016), in a community based prospective study, reported that the risk of dyslipidemia in women with menstrual irregularities was similar to a healthy population (OR= 1.00, 95% CI: 0.76–1.31) (66). Further, no associations between PCOM and metabolic disorders were found in the same setting (26). Besides, we found that serum level of TT among women with PCOM was higher than healthy population, but those increased androgen values did not meet the hyperandrogenemia. Likewise, Chang et al. (2000) suggested that increased secretion of LH and FSH in response to stimulation of Gonadotropin hormone-releasing hormone (GnRH) by agonists may lead to increased serum concentration of ovarian androgens among women with PCOM compared with controls (67).

Some bodies of literature support the association between PCOS, obesity, IR and metabolic disorders (4, 68, 69). Besides, adiposity indicators may be markers that show energy metabolism changes. It *per se* could influence the risk of cardio-metabolic disorders and mortality. BMI, a simple indicator of obesity, is the most commonly used index to characterize obesity in individuals. However, it is well documented that BMI *per se* is a risk factor for cardio-metabolic disorders (70); therefore, to minimize or avoid any bias, we adjusted this indicator in our analyses. The adiposity indicators of VAI and LAP include both anthropometric, and lipid parameters and have been proposed as valuable indicators of visceral adipose function (71). They are known to reliably predict IR, MetS, cardiovascular events and all-cause mortality in non-diabetic patients (72, 73). Our study showed that both VAI and LAP values in women with PCOS, particularly in severe phenotypes of PCOS, are significantly higher than in healthy women. However, since both VAI and LAP are indicators based on TG, we hypothesized that a higher level of these indicators might highly correlate with higher level of TG in severe phenotypes of PCOS. Some studies supported these findings that showed the higher values of VAI and LAP among women with PCOS compared to non-PCOS counterparts (74–76).

Emerging evidence suggests that ovarian volume ≥10 ml appears to be a good surrogate indicator of PCOM when the older imaging technology (probes with a maximum frequency of 5 MHz) is used (77, 78). However, since this recommendation is not included in the diagnostic criteria derived from the Rotterdam criteria, we used the original PCOM definition.

This study has its strengths, such as using a population-based sample instead of recruitment from a tertiary center. In addition, we used national cut-points of androgens hormones for the definition of hyperandrogenism. Adjustment for potential confounders helped us to present more reliable results. To mention the limitations, for virgin cases, socio-cultural constraints did not let us to perform a transvaginal ultrasonography determining the polycystic ovaries.

In conclusion, the results of our study revealed that PCOS, mainly classical phenotypes A and B, are common among Iranian women of reproductive age. Moreover, one-third of Iranian reproductive-aged women suffered from only one of the criteria for PCOS. In addition, women with PCOS who had androgen excess exhibited the worst lipid profile and those who had all three components of the syndrome showed the higher rate of MetS. However, women with only ovulatory dysfunction and only PCOM had similar cardio-metabolic characteristics, when compared to healthy subjects. These data suggest that routine screening for metabolic disturbances may be needed in the prevention of cardio-metabolic disorders in patients with more serious phenotypes of PCOS.

## Data Availability Statement

The raw data supporting the conclusions of this article will be made available by the authors, without undue reservation.

## Ethics Statement

The studies involving human participants were reviewed and approved by the ethical review board of the Research Institute for Endocrine Sciences (Code: IR.SBMU.ENDOCRINE.REC.1398.070). The patients/participants provided their written informed consent to participate in this study.

## Author Contributions

MF-A, MR, SB-G, and FRT contributed to the conception and planning. MF-A, FM, FRT, and EKP contributed to carrying out the study. SB-G, MR, MF-A, FA, and FRT were involved in data analysis and drafted the article. All authors listed have made a substantial, direct, and intellectual contribution to the work and approved it for publication.

## Conflict of Interest

The authors declare that the research was conducted in the absence of any commercial or financial relationships that could be construed as a potential conflict of interest.

## Publisher’s Note

All claims expressed in this article are solely those of the authors and do not necessarily represent those of their affiliated organizations, or those of the publisher, the editors and the reviewers. Any product that may be evaluated in this article, or claim that may be made by its manufacturer, is not guaranteed or endorsed by the publisher.
