# Supplementary material for: The Prevalence of Polycystic Ovary Syndrome, Its Phenotypes and Cardio-Metabolic Features in a Community Sample of Iranian Population: Tehran Lipid and Glucose Study
Source: Front Endocrinol (Lausanne). 2022 Mar 1;13:825528. doi: 10.3389/fendo.2022.825528 (PMC8920974; doi:10.3389/fendo.2022.825528)
Supplement: Supplementary file 1 [file DataSheet_1.docx]

**Supplementary file:**

**Anthropometric and Biochemical Measurement**

All information about demographic variables, obstetrics and reproductive history were gathered by a standard comprehensive questionnaire during face-to-face interviews. In addition, the general anthropometric and physical examinations included an assessment of hirsutism using the modified Ferriman-Gallwey scoring method (29), performed by a general practitioner. Weight and height were measured in the standing position, with participants wearing minimal clothing by trained staff using standardized procedures and calibrated equipment.

Blood pressure was measured using a digital sphygmomanometer with an appropriate sized cuff for arm diameter in a supine position after 15 min rest. Transvaginal or transabdominal ovaries ultrasound of the study participants performed using the 3.5-MHz transabdominal and 5-MHz transvaginal transducer by an experienced sonographer. Ultrasound was performed on the same day as the blood samples were collected.

All blood samples were taken between 7:00 and 9:00 AM after 12 hours of overnight fasting during the early follicular phase of the spontaneous or progesterone-induced menstrual cycle. All sera were stored at -80°C until the time of measurements. Luteinizing hormone (LH) and Follicle-stimulating hormone (FSH) was measured by immunoradiometric assay (IRMA) (Izotop, Budapest, Hungary) using gamma counter Wallac Wizard, Turku, Finland). 17-hydroxyprogesterone (17OH-P), Total testosterone (TT), Androstenedione (A4) and Dehydroepiandrosterone sulfate (DHEAS), were measured by enzyme immunoassay (EIA) (Diagnostic biochem Canada Co. Ontario, Canada). Sex Hormone Binding Globulin (SHBG) was measured by immunoenzymometric assay (IEMA), (Mercodia, Uppsala, Sweden). Free androgen index (FAI) was calculated using the formula [TT (nmol/L) × 100/SHBG (nmol/L)]. All ELISA tests were performed using Sunrise ELISA reader (Tecan Co. Salzburg, Austria). The intra- and inter-assay coefficients of variation (CVs) for LH were 3% and 5.8%; for FSH: 3.5% and 4%; for 17 OH-P: 4.8% and 6.8%; for TT: 5.6% and 6.6%; for A_4_: 2.2% and 3.5%; for DHEAS: 2.0% and 5.1%; and for SHBG: 1.2% and 5.7%, respectively.

Fasting blood glucose (FBG) was assessed using glucose oxidase by enzymatic colorimetric method (Pars Azmoon kit, Iran, intra- and inter-assay < 2.2%). Total cholesterol (TC) was measured (enzymatic colorimetric method with cholesterol esterase and cholesterol oxidase). HDL-C was assayed after precipitation of the apolipoprotein B (apo B)-containing lipoproteins with phosphotungstic acid. TG was assayed using glycerol phosphate oxidase. Intra- and inter-assay coefficients of variations for TC, HDL-C, and TG were below 1.9, 3, and 2.1%, respectively. Analyses were performed using related kits (Pars Azmon Inc., Tehran, Iran) and a Selecta 2 autoanalyzer (Vital Scientific, Spankeren, Netherlands). To calculate LDL-C, the modified Friedewald equation was used (30). The intra- and inter-assay CVs were both 2.2% for glucose. Intra- and inter-assay CVs were 0.6% and 1.6% for TG, respectively. For both total and HDL-C, intra- and inter-assay CVs were 0.5% and 2%, respectively (31)**.**
